# p38 MAPK and JNK Antagonistically Control Senescence and Cytoplasmic p16INK4A Expression in Doxorubicin-Treated Endothelial Progenitor Cells

**DOI:** 10.1371/journal.pone.0015583

**Published:** 2010-12-20

**Authors:** Paolo Spallarossa, Paola Altieri, Chiara Barisione, Mario Passalacqua, Concetta Aloi, Giuseppina Fugazza, Francesco Frassoni, Marina Podestà, Marco Canepa, Giorgio Ghigliotti, Claudio Brunelli

**Affiliations:** 1 Division of Cardiology, Research Center of Cardiovascular Biology, University of Genova, Genova, Italy; 2 Biochemistry Section, Department of Experimental Medicine, Centre of Excellence for Biomedical Research, University of Genova, Genova, Italy; 3 Laboratory of Cytogenetics, Department of Internal Medicine, University of Genova, Genova, Italy; 4 Stem Cell and Cell Therapy Centre, San Martino Hospital, Genova, Italy; 5 2nd Division, Department of Hematology, S. Martino Hospital, Genova, Italy; Istituto Dermopatico dell'Immacolata, Italy

## Abstract

Patients treated with low-dose anthracyclines often show late onset cardiotoxicity. Recent studies suggest that this form of cardiotoxicity is the result of a progenitor cell disease. In this study we demonstrate that Cord Blood Endothelial Progenitor Cells (EPCs) exposed to low, sub-apoptotic doses of doxorubicin show a senescence phenotype characterized by increased SA-b-gal activity, decreased TRF2 and chromosomal abnormalities, enlarged cell shape, and disarrangement of F-actin stress fibers accompanied by impaired migratory ability. P16^ INK4A^ localizes in the cytoplasm of doxorubicin-induced senescent EPCs and not in the nucleus as is the case in EPCs rendered senescent by different stimuli. This localization together with the presence of an arrest in G2, and not at the G1 phase boundary, which is what usually occurs in response to the cell cycle regulatory activity of p16^INK4A^, suggests that doxorubicin-induced p16^ INK4A^ does not regulate the cell cycle, even though its increase is closely associated with senescence. The effects of doxorubicin are the result of the activation of MAPKs p38 and JNK which act antagonistically. JNK attenuates the senescence, p16^ INK4A^ expression and cytoskeleton remodeling that are induced by activated p38. We also found that conditioned medium from doxorubicin-induced senescent cardiomyocytes does not attract untreated EPCs, unlike conditioned medium from apoptotic cardiomyocytes which has a strong chemoattractant capacity. In conclusion, this study provides a better understanding of the senescence of doxorubicin-treated EPCs, which may be helpful in preventing and treating late onset cardiotoxicity.

## Introduction

The clinical use of doxorubicin, the most widely used anthracycline, is limited by its cardiotoxic effects which include cardiomyopathy and heart failure [1]. The most likely hypothesis regarding doxorubicin cardiotoxicity is that doxorubicin induces cardiomyocyte loss through oxidative stress and apoptotic cell death [2]. Recent studies have shown that doxorubicin targets mature differentiated cardiomyocytes as well as progenitor cells including cardiac progenitor cells that are primitive, proliferating, resident cells which regenerate myocytes and vessels in vivo, and bone marrow derived endothelial progenitor cells (EPCs) that are mobilized to peripheral circulation and home to sites of myocardial injury [3]–[5]. Inhibition of the progenitor cell mediated self-repairing potential of the heart is now considered a major pathogenetic mechanism of doxorubicin-induced cardiomyopathy.

A second emerging concept related to anthracycline cardiotoxicity concerns the type of cell damage caused by these drugs. Recent studies have shown that doxorubicin toxicity is dose-dependent. At high doses it induces apoptosis, while at low doses it causes a phenomenon known as stress-induced premature senescence (SIPS) which is the result of changes in the expression of many proteins that regulate cell cycle, cytoskeletal and cellular architecture, and that impair cell function and may lead to late death [6], [7]. To our knowledge no experimental evidence has been reported regarding doxorubicin-induced senescence in EPCs. Thus, the aim of the present study was to assess the vulnerability of EPCs to doxorubicin-induced senescence by examining both structural and functional changes. To investigate the pathways of this process, we focused on telomere dysfunction, mitogen-activated protein kinases (MAPK) p38 and c-Jun N-terminal kinases (JNK), and p16^INK4A^ which have proven to play a role in both doxorubicin-induced senescence in cardiomyocytes and in senescence in EPCs induced by other types of stress.

## Materials and Methods

All materials, unless otherwise indicated, were supplied by Sigma-Aldrich (Poole, UK).

### Cell and culture conditions

Cord Blood (CB) EPCs were obtained from mononuclear cells isolated from human umbilical CB samples obtained from the Cord Blood Bank. Cells were isolated, cultured and characterized as described by Corselli et al [8] and used from passage 2 to 5 at less than 75% of confluence.

Approval from the Ethics Committee of our institution was not necessary because CB cells were obtained in compliance with Italian legislation and mothers gave informed written consent to cord donation, cell banking and utilization for research purpose in case of sample with cell content numerically unsuited for clinical use.

### Experimental design

Cells were pre-incubated for 1 hour with or without the JNK inhibitor SP600125 (20 µmol/L (Calbiochem, Merck KgaA, Darmstadt, Germany), and/or the p38 inhibitor SB203580 (3 µmol/L) (Calbiochem, Merck KgaA, Darmstadt, Germany). They were then incubated with or without various doses of doxorubicin for 3 hours [9] and analyzed at the time indicated for each experiment. Since the MAPK inhibitors were dissolved in 0.1% dimethyl sulphoxide (DMSO), an equivalent amount of vehicle was added to both the control and to the drug-treated samples. Twenty-four hours after the end of the treatments, cells were analyzed for proliferation [Bromodeoxyuridine, BrdU, incorporation and 3-(4,5-dimethylthiazol-2-yl)-2,5-diphenylterazolium bromide (MTT) assay], apoptosis (single-stranded DNA, ssDNA), Annexin V–fluorescein isothiocyanate (FITC)/propidium iodide staining (AV/PI), F-actin, and immunoblotting. Forty-eight hours after the end of the treatments cells were analyzed for senescence, metaphases, cell cycle immunofluorescence and immunohistochemistry.

We chose to analyze the level of senescence at this time point because had it been analyzed earlier, the rate of senescent cells would have been too low to allow statistical analysis, while in case of longer culture times, untreated EPCs would have spontaneously undergone replicative senescence.

### Total cell lysates

Cells were lysed in lysis buffer [20 mM Tris HCl (pH 7.5), 150 mM NaCl, 1 mM Na_2_EDTA, 1 mM EGTA, 1% NP40, 2.5 mM Na_2_P_2_O_7_, and 1 mM β-glycerophosphate]. The following inhibitors were added immediately before buffer was added to cells: 1 mM phenylmethylsulfonyl fluoride, 1 mM Na_3_VO_4_, 1 mM NaF, and protease inhibitor mixture (Roche).

### Subcellular fractionation

Cytoplasmic and nuclear fractions were obtained as previously described [10].

### Immunoblotting

Immunoblotting was performed using the previously described procedure [9]. After various treatments, cells were processed to determine the levels of TRF2 (clone 4S794.15, Imgenex, San Diego, CA), p16^INK4A^ (N-20), GAPDH (0411), phosphorylated p38 (E-1) (ph-p38), p38 (H-147), phosphorylated JNK (G-7) (ph-JNK) and JNK (FL) (Santa Cruz Biotechnology, Santa Cruz, CA). ph-p38 and ph-JNK levels were evaluated at each time-point indicated in the experiment**s**. After incubation in horseradish peroxidase secondary antibody, blots were visualized with ECL substrate (Amersham Bioscences, NJ) and films were quantified by densitometry with an image analyzer system (Syngene,UK). Filters were stripped and reprobed with GAPDH in order to normalize the amounts of TRF2 and p16, and with p38 and JNK antibodies to normalize the amounts of ph-p38 and ph-JNK respectively.

### SA-b-gal activity (Senescence-associated -β-Galactosidase Staining)

Cells were stained for β-galactosidase activity as described by Dimri [11].

The ability to induce SA-b-gal activity is a manifestation of residual lysosomal activity at suboptimal pH (pH 6). It becomes detectable in the course of senescence because of the increased lysosomal content on senescent cells [12]. The number of SA-b-gal positive cells was determined in 100 randomly chosen low-power fields (x100) and expressed as a percentage of all counted cells.

### Immunocytochemistry

The expression of p16^INK4A^ protein was documented by immunostaining using the procedure described elsewhere [13]. Cells were examined by light microscopy for image analysis.

### Confocal microscopy

Cells were fixed and permeabilized with 4% paraformaldehyde/0.1% Triton X-100 for 10 minutes at 4°C immediately before being processed for immunofluorescence. Non-specific antibody binding was blocked by a 30 minute incubation period with 5% (v/v) fetal calf serum. Cells were then treated with 2 µg/ml rabbit anti p16^INK4A^ (Santa Cruz biotechnology, Inc., Santa Cruz, California, USA), followed by an Alexa Fluor488 anti-rabbit secondary antibody (Invitrogen). Nuclei were identified by PI staining. Images were taken using a Leica TCS SL2 confocal microscope (Leica Wetzlar, Germany) equipped with argon/He-Ne laser sources and an HCX PL APO CS 63.0×1.40 oil objective. Excitation and emission wavelengths were 488 and 522 nm for the Alexa-labeling antibody and 543 and 605 nm for PI staining, respectively.

### BrdU assay

BrdU, the thymidine analog that is incorporated into the DNA of dividing cells during S-phase, was used for mitotic labeling. EPC-derived cells were grown on a chamber slide and treated with or without various doses of doxorubicin for 3 h. After 24 hours, cells were incubated with BrdU (10 µM) for 1 h, fixed in 70% ethanol, incubated with an anti-BrdU FITC antibody (Becton Dikinson, San Jose, CA) for 30 minutes and observed under a fluorescence microscope [14].

### MTT Assay

The assay, which is based on the reduction of the tetrazolium salt MTT by active mitochondria to produce insoluble formazan salt, measures mitochondrial metabolic activity and is often used as indicator of cell viability. Cells were treated in 96-well plates, MTT was added to each well under sterile conditions (final concentration of 5 mg/ml), and the plates were incubated for 3 h at 37°C. Formazan crystals were dissolved in dimethyl sulfoxide (100 ml/well). The purple formazan crystals were formed from yellow MTT by succinate dehydrogenase in viable cells. Absorbance of the formazan product was measured at 570 with a background correction at 690 nm using a microplate reader [15].

### Annexin V–fluorescein isothiocyanate (FITC)/propidium iodide staining

Cells were labeled with AV/PI, and 100 randomly selected fields were counted using a fluorescence microscope. The number of stained cells was normalized to the total number of cells as counted by phase contrast microscopy of the same field.

### Detection and quantitation of apoptosis by ssDNA antibodies

Cell mono layers grown on slides were exposed to various doses of doxorubicin, and apoptotic cells were identified by ssDNA immunostaining as previously described [16]. The number of stained cells was normalized to the total number of cells as counted by fluorescence microscope of the same field.

### Flow cytometric analysis

Cells for cell cycle analysis were maintained in complete medium (EGM-2-MV, Lonza, Walkersville, MD). Trypsinized and floating cells were pooled, washed twice with PBS and resuspended in 400 µl of hypotonic labeled solution, 5 µg/ml PI, 0.1% w/v Na citrate, 0.1%Triton X-100 in sterile water. Cells were incubated on ice for 30 min until DNA content analysis.

**N**uclear DNA content and cell cycle analysis were monitored by Fluorescence Activated Cell Sorting (Becton Dickinson, San Jose, CA) and data were analyzed using CellQuest software (Becton Dickinson San Jose, CA).

### Chromosome analysis

Twenty-four hours after treatment with doxorubicin, cells were exposed to Colcemid (0.04 µg/ml) for 90 minutes at 37°C, and to hypotonic treatment (0.075 mol/L KCl) for 15 minutes at room temperature. Cells were fixed in a methanol and acetic acid (3∶1 by volume) mixture for 15 minutes and then washed three times in the fixative. The slides were air-dried and stained with Giemsa for analysis.

### F-actin detection

EPCs growing on slides were fixed, permeabilized and labeled simultaneously in PBS containing 50 µg/ml lysopalmitoyphospatidylecholine, 3.7% formaldehyde and 5 units/ml of fluorescent phallotoxin (A-12379 Alexa™488 phalloidin, Molecular probes, Inc). Cells were rapidly washed three times with PBS and were viewed by fluorescent microscopy in order to carry out image analysis [17].

### EPC migration assay

EPC migration was assayed using a modified Boyden chamber composed of a membrane with an 8 µm pore size.

#### EPC migration after treatment with doxorubicin

After treatments with doxorubicin EPCs (10^4^ cells per well) were seeded onto chemotaxis filters in serum free EBM plus 0.1% bovine serum albumin (BSA). EGM-2-MV containing VEGF (50 ng/ml) was then added to the lower chamber.

#### EPC migration with conditioned medium from H9c2

EPCs (10^4^ cells per well) were seeded onto chemotaxis filters in serum free EBM plus 0.1% bovine serum albumin. The following were then added to the lower chamber: DMEM plus 0.1% BSA, conditioned media from H9c2 cardiomyocytes, and EGM-2-MV containing VEGF (50 ng/ml). In order to obtain conditioned medium from senescent or apoptotic H9c2, we treated the cells for 3 hours with 0.1 or 1 µM doxorubicin, respectively, in growth factor-free DMEM plus 0.1% BSA, then the H9c2 were grown in fresh medium (growth factor-free DMEM plus 0.1% BSA) for 24 hours [9]. DMEM plus 0.1% BSA served as the control medium and EGM-2-MV containing VEGF served as the medium of activation.

After the 24-hour migration period, the lower side of the nitrocellulose membrane was washed with PBS and fixed with 2% paraformaldehyde. Cells were stained with Giemsa solution for quantification. Migrating cells were counted manually in four random microscopic fields [18].

### Image analysis

Image analysis was performed by the Leica Q500 MC Image Analysis System (Leica, Cambridge, UK). Three hundred cells were randomly analyzed for each sample**,** and the optical density of the signals was quantitated by a computer. The video image was generated by a CCD Camera connected through a frame grabber to a computer. Single images were digitized for image analysis at 256 grey levels. Imported data were quantitatively analyzed by Q500MC Software-Qwin (Leica, Cambridge, UK). The single cells were randomly selected by the operator by using the cursor and then positive areas were automatically estimated.

### Statistical analysis

Data are reported as mean ± standard error (SE) of four independent experiments. Statistical analysis was performed by one-way ANOVA followed by Bonferroni post-hoc test and Wilcoxon signed rank test when appropriate.

## Results

### Low and high doses of doxorubicin induce EPC senescence and apoptosis

**Figure 1:**
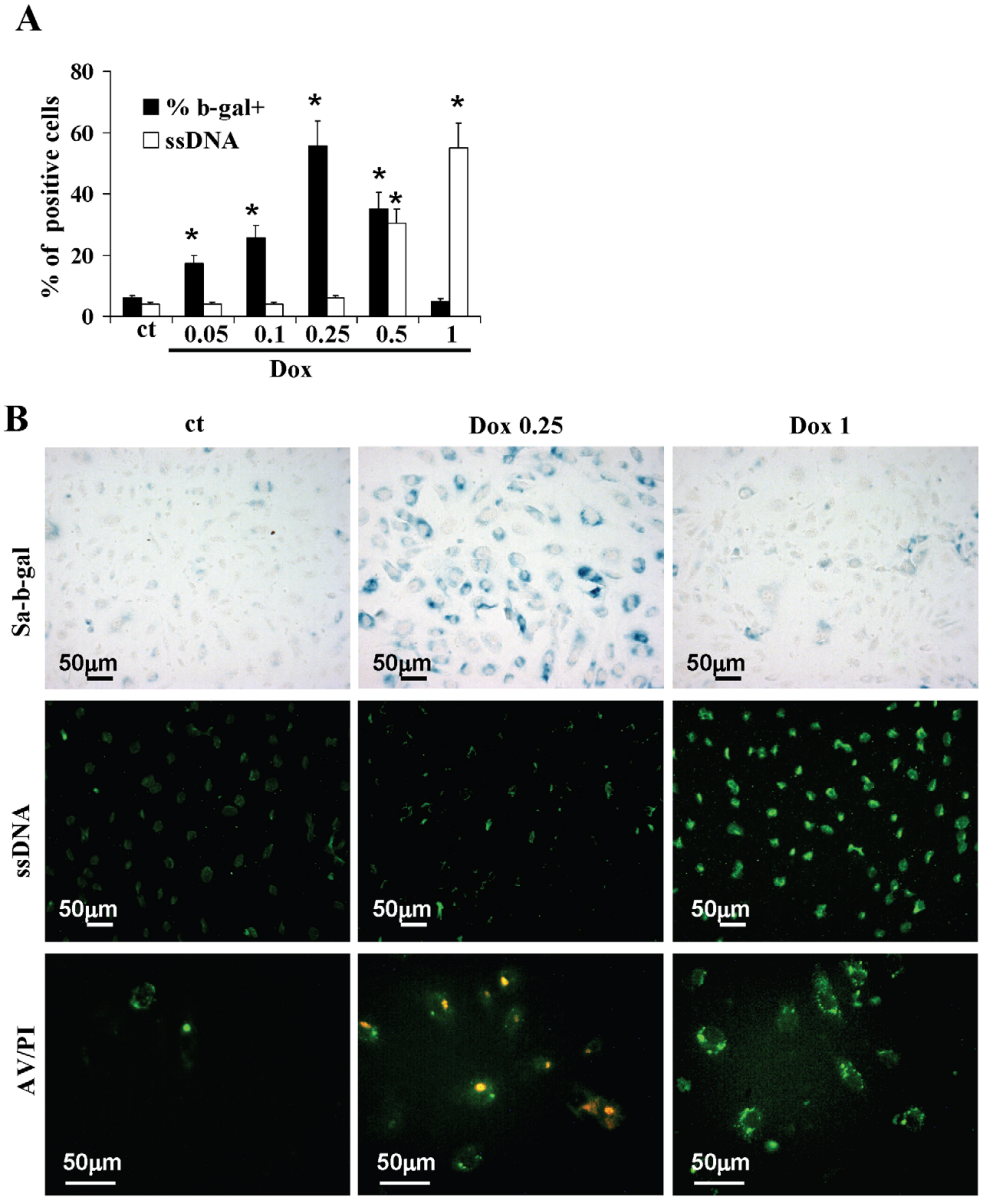
Dose-effect curve. (A) Bar-graph showing the percentages of SA-b-gal and ssDNA positive cells after treatment with various doses of doxorubicin. (B) Photographs illustrating the effects of doxorubicin 0.25 and 1 µM. Cells were evaluated for (from top to bottom): SA-b-gal activity (magnification, ×200), ss-DNA positivity (magnification, ×200), and AV/PI staining (magnification, ×400). ct, control; Dox, doxorubicin. *p<0.05 vs control.

To investigate the effects of doxorubicin on senescence and apoptosis, we performed a dose-effect curve. At doses of 0.05, 0.1 and 0.25 µM, doxorubicin did not increase the percentage of ssDNA positive cells, while it did induce a dose-dependent increase of SA-b-gal positivity accompanied by a flattened and enlarged cell shape, thus suggesting a pro-senescent non apoptotic effect. (Figure 1A, 1B). At doses of 0.5 and 1 µM, doxorubicin had a pro-apoptotic effect documented by a dose-dependent increase in ssDNA positivity and decrease in SA-b-gal positivity. AV/PI staining showed that cells treated with doxorubicin 1 µM were AV(+)/PI(-) thus showing the typical features of early apoptosis, while cells treated with 0.25 µM were AV(+)/PI(+) (Fig. 1B). Even though this double positivity might indicate late apoptosis or necrosis, taking into account the results of ssDNA and SA-b-gal analysis and in agreement with previous studies performed by others [19] and by ourselves [7], [16], this result suggests that sub-apoptotic doses of doxorubicin induce early loss of membrane permeability in this experimental model.

In further experiments we chose the doses of 0.25 µM and 1 µM doxorubicin to induce senescence or apoptosis respectively.

### Doxorubicin induces the increase of p16^INK4A^ with perinuclear accumulation

**Figure 2:**
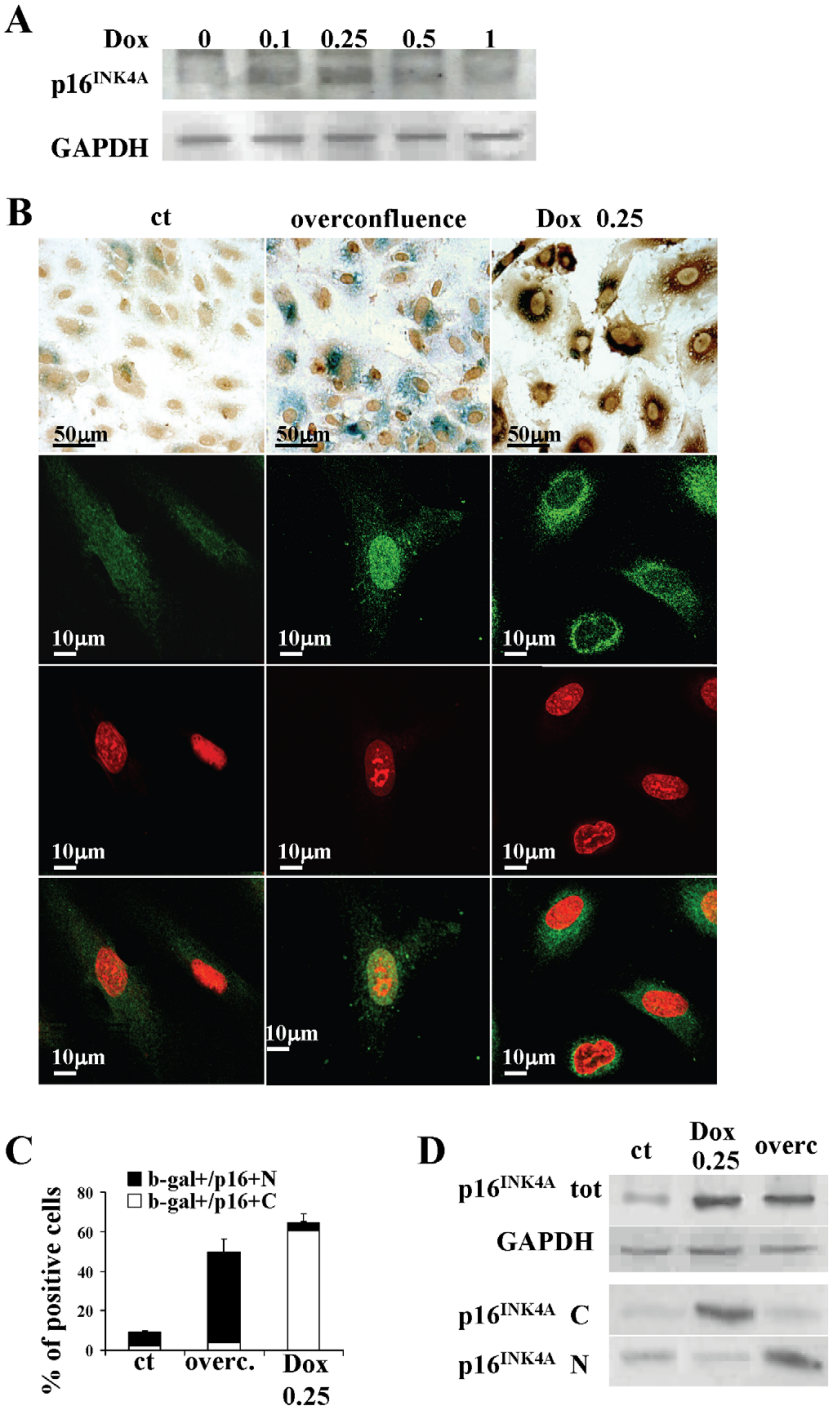
Prosenescent doses of doxorubicin increase p16^INK4A^ protein levels with cytoplasmic accumulation. (A) Western blot analysis of p16^INK4A^ expression in EPCs treated with various doses of doxorubicin. (B) Photographs show from left to right: control cells, cells cultured for four days after confluence (overconfluence), cells treated with 0.25 µM doxorubicin; first row: merge of SA-b-gal staining (blue) and immunocytochemistry for p16^INK4A^ (brown) (magnification, ×400)); second row, p16^INK4A^ immunofluorescence (magnification, ×1000); third row, nuclear staining with PI (magnification, ×1000); fourth row, merge of p16^INK4A^ immunofluorescence and nuclear staining with PI (magnification, ×1000).(C) Bar-graph showing the percentage of SA-b-gal positive cells having nuclear (SA-b-gal+/p16+N) or cytoplasmic p16^INK4A^ localizations (SA-b-gal+/p16+C) in control, overconfluent for four days or 0.25 µM doxorubicin treated cells. (D) Western blot analysis of p16^INK4A^ expression in EPCs treated with 0.25 µM doxorubicin or cultured for four days after confluence. ct, control; Dox, doxorubicin; overc., overconfluence; tot, total; N, nuclear; C, cytoplasmic.

We also evaluated the effects of doxorubicin on p16^INK4A^, a cyclin-dependent kinase inhibitor thought to be a senescence-associated marker. Western blot analysis documented that untreated EPCs express very low levels of p16^INK4A^ (Fig. 2A). A dose effect curve shows that doxorubicin induces changes in p16^INK4A^ expression levels that parallel changes in the percentage of Sa-b-gal positive cells. Immunocytochemical analysis of p16^INK4A^ merged with SA-b-gal activity together with confocal microscopy analysis of p16^INK4A^ revealed that, in cells treated with 0.25 µM doxorubicin, p16^INK4A^ is increased in SA-b-gal positive cells and that it has cytoplasmic, perinuclear localization (Fig. 2B, 2C,). We also evaluated p16^INK4A^ in a model of EPC senescence not induced by doxorubicin [20]. After having maintained EPCs in culture for four days after confluence, we observed a significant increase in p16^INK4A^ expression levels with typical nuclear localization (Fig. 2B, 2C,). Western analysis of total, cytoplasmic and nuclear protein extracts indicated that 0.25 µM doxorubicin induces p16^INK4A^ accumulation in the cytosol while overconfluence induces p16 accumulation in the nucleus (Fig. 2D).

### P38, JNK and oxidative stress regulate induction of senescence and p16 INK4A

**Figure 3:**
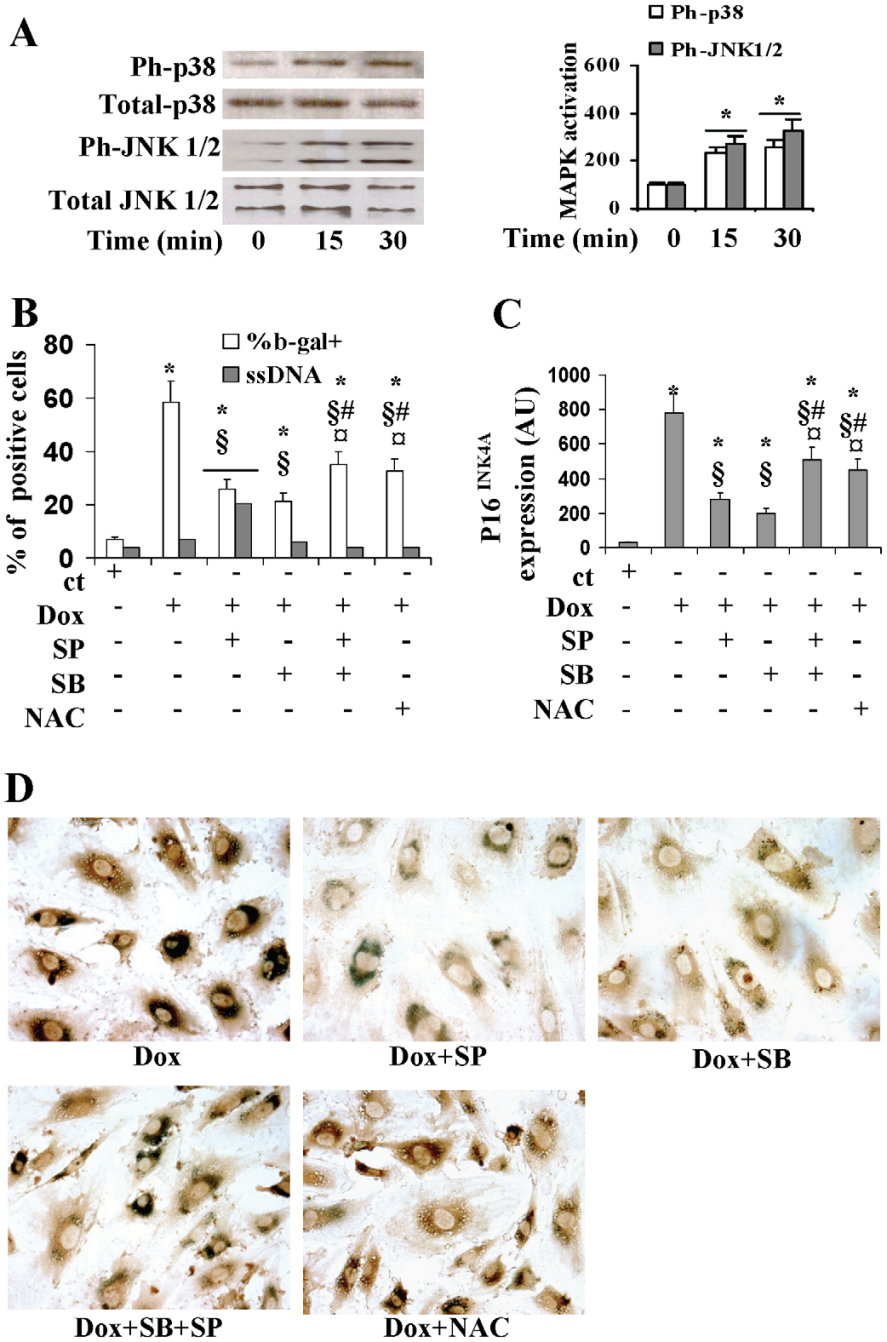
p38 and JNK control the cell response to sub-apototic doses of doxorubicin differently. (A):effects of exposure to 0,25 µM doxorubicin on MAPK activation by western blot using antibodies specific for phosphorilated (Ph) and total p38 and JNK (left panel). Bar graph showing values for ph-MAPK normalizzated to the amount of total enzyme and expressed as the relative increase above control value, which was set at 100 (right panel). (B) and (C): bar-graphs showing the effects of pre-treatment with the JNK inhibitor, SP600125, the p38 inhibitor, SB203580 and NAC on the percentage of SA-b-gal and ssDNA positive cells (panel B) and p16^INK4A^ protein levels (panel C). (D): merge of SA-b-gal staining (blue) and immunocytochemistry for p16^INK4A^ (brown) in cells exposed to doxorubicin and pre-treated with or without SP600125, SB203580, or NAC (magnification, ×400)**.** ct, control; Dox, 0.25 µM doxorubicin. SB, p38 inhibitor; SP, JNK inhibitor. *p<0.05 vs ct; § p<0.05 vs Dox.; ¤ p<0.05 vs. SP+Dox; # p<0.05 vs. SB+Dox.

As demonstrated by previous studies, doxorubicin acts through the activation of MAPKs and the induction of oxidative stress. We demonstrated that the p38 and JNK pathways are activated by 0.25 µM doxorubicin, as evidenced by phosphorylation of p38 and JNK. We found no changes in total MAPK protein levels (Fig. 3A). We then analyzed the role played by MAPKs in mediating the effect of doxorubicin on senescence and p16INK4A induction. Pre-treatment of EPCs with SB203580, a p38 specific inhibitor, or SP600125, a JNK specific inhibitor, attenuated the doxorubicin-induced increase of SA-b-gal positivity (−63% and −71% respectively) and of p16INK4A protein levels (−66% and −77% respectively) (Fig 3B, 3C). Pre-treatment with both inhibitors, i.e., SB203580 and SP600125, reduced the increase of SA-b-gal positivity by 45% and the increase of p16^ INK4A^ expression levels by 44%, thus resulting less effective than pre-treatment with the individual inhibitors alone (Fig. 3B, 3C). As shown in Figure 3D, pre-treatment with these inhibitors did not influence the p16^ INK4A^ localization which remained cytoplasmatic and perinuclear. Pre-treatment with PD98059, an ERK 1/2 specific inhibitor, did not modify the effects induced by doxorubicin (data not shown). Pre-treatment for 90 minutes with the antioxidant NAC (10 µM) reduced the doxorubicin induced increase of SA-b-gal positivity by 49% and of p16INK4A expression by 56% (Fig. 3B, 3C, 3D).

We then analyzed the effects of JNK and p38 inhibitors on ssDNA positivity. Pre-treatment with SP600125 of cells exposed to the sub-apoptotic dose of 0.25 µM doxorubicin significantly increased the percentage of ssDNA positive cells (20% vs 5%), while pre-treatment with SB203580 did not produce any effects (Fig. 3B). These results collectively show that while pre-treatment with the specific p38 inhibitor mitigates the toxic effects of doxorubicin, pre-treatment with the specific JNK inhibitor decreases senescence but induces apoptosis.

### F-actin disorganization induced by Doxorubicin is p38 and JNK mediated

**Figure 4:**
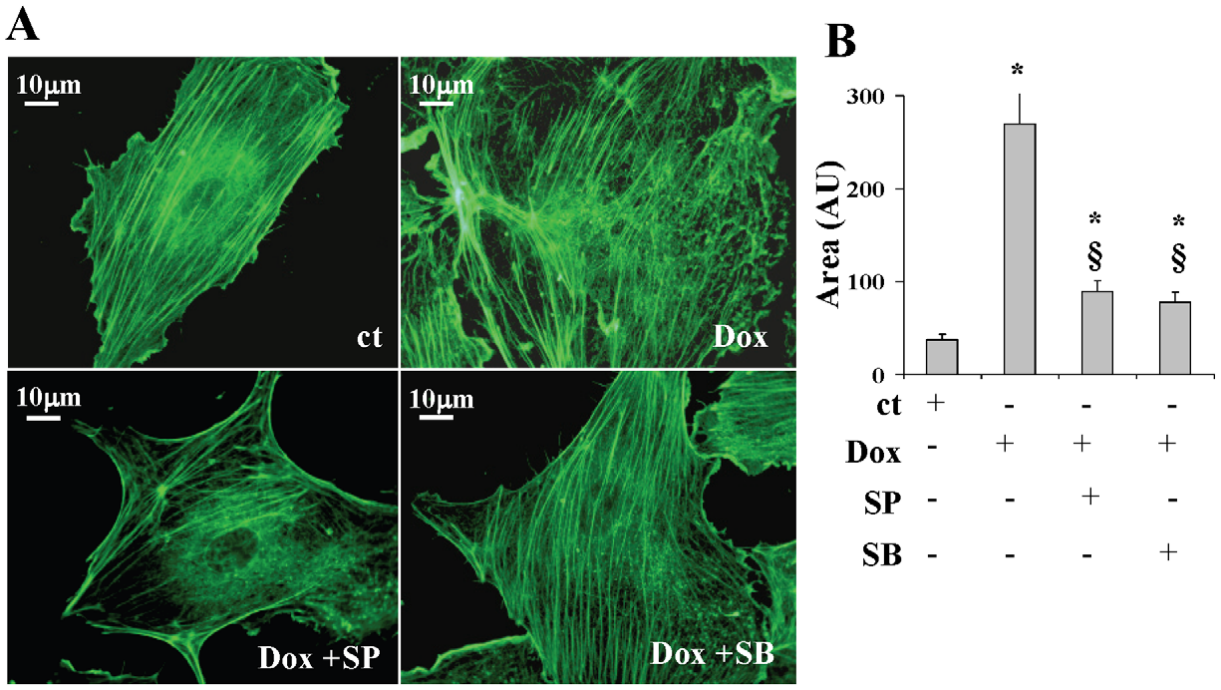
Phalloidin staining of F-actin. (A) Photographs depict EPCs pre-treated with or without MAPK inhibitors, and then treated with 0.25 µM doxorubicin (magnification 1000X). (B) Bar-graph showing cell size in various treatment groups. ct, control; Dox, 0.25 µM doxorubicin. SB, p38 inhibitor; SP, JNK inhibitor. *p<0.05 vs ct; § p<0.05 vs Dox.

F-actin is well organized in linear stress fibers in normal EPCs. After treatment with Doxorubicin 0.25 µM, cells appeared enlarged, without detectable changes in the total amount of F-actin stress fibers, which, however, resulted disorganized, partially destroyed, and thickened at the cell periphery (Fig. 4A, 4B). Pre-treatment with p38 or JNK inhibitors reduced hypertrophy, but while SB203580, the p38 inhibitor almost completely restored the normal structure of the cytoskeleton, SP600125, the JNK inhibitor, led to the disappearance of F-actin fibers. This confirms that JNK inhibition switches cell response to doxorubicin 0.25 µM (Fig. 4A, 4B) from senescence to apoptosis [21].

### Effect of doxorubicin on EPC migration

**Figure 5:**
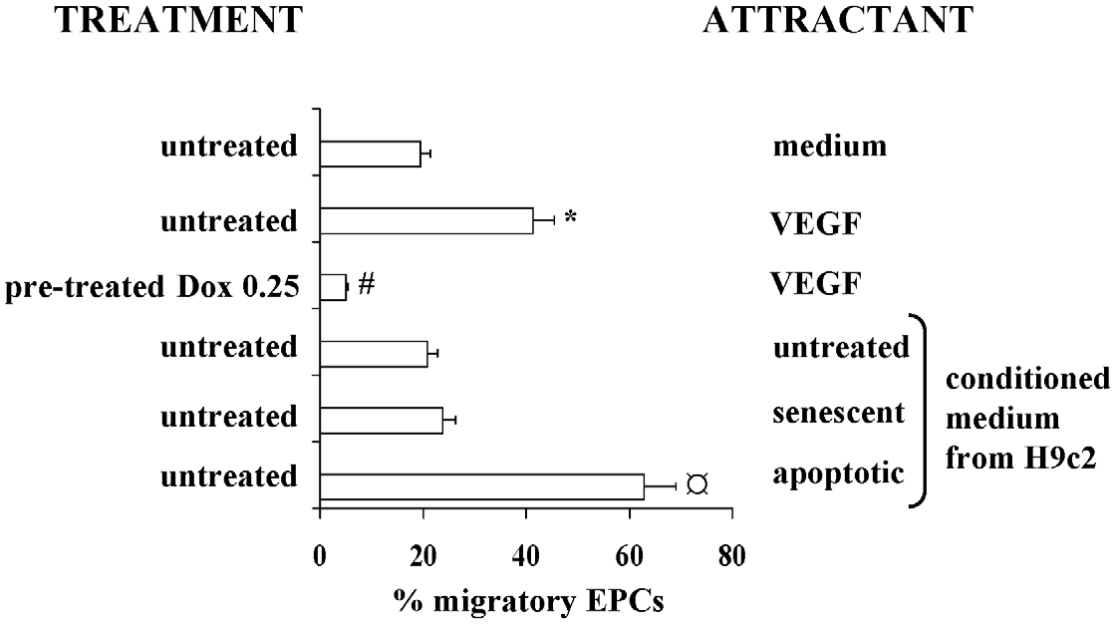
EPC migration assay. Graph represents the percentage of migrating EPCs assessed by a modified Boyden chamber assay; EPCs were untreated or incubated with 0.25 µM doxorubicin and then exposed to: VEGF, medium, or conditioned medium from normal or doxorubin-induced senescent or apoptotic H9c2 cells. ct, control; Dox, doxorubicin; SP, JNK inhibitor; SB, p38 inhibitor; ct, control; *p<0.05 vs medium; # p<0.05 vs untreated cells exposed to VEGF; ¤ p<0.05 vs. all conditions.

Doxorubicin-induced cytoskeleton remodelling suggests that doxorubicin may alter the migratory ability of EPCs. The modified Boyden chamber assay demonstrated that the migratory capacity of EPCs was lost in doxorubicin-treated cells (Fig. 5).

### Effect of apoptotic and senescent cardiomyocytes on EPC migration

The capacity of damaged tissue to recruit EPCs is a key event in the process of tissue repair. We therefore investigated the effect of conditioned medium from doxorubicin-induced apoptotic or senescent H9c2 cardiomyocytes on EPC migration. Conditioned medium from apoptotic cardiomyocytes stimulated EPC migration, while conditioned medium from senescent cardiomyocytes did not produce any effect (Fig. 5).

### Prosenescent doses of doxorubicin decrease TRF2 levels and induce anomalous mitoses and cell cycle alterations

**Figure 6:**
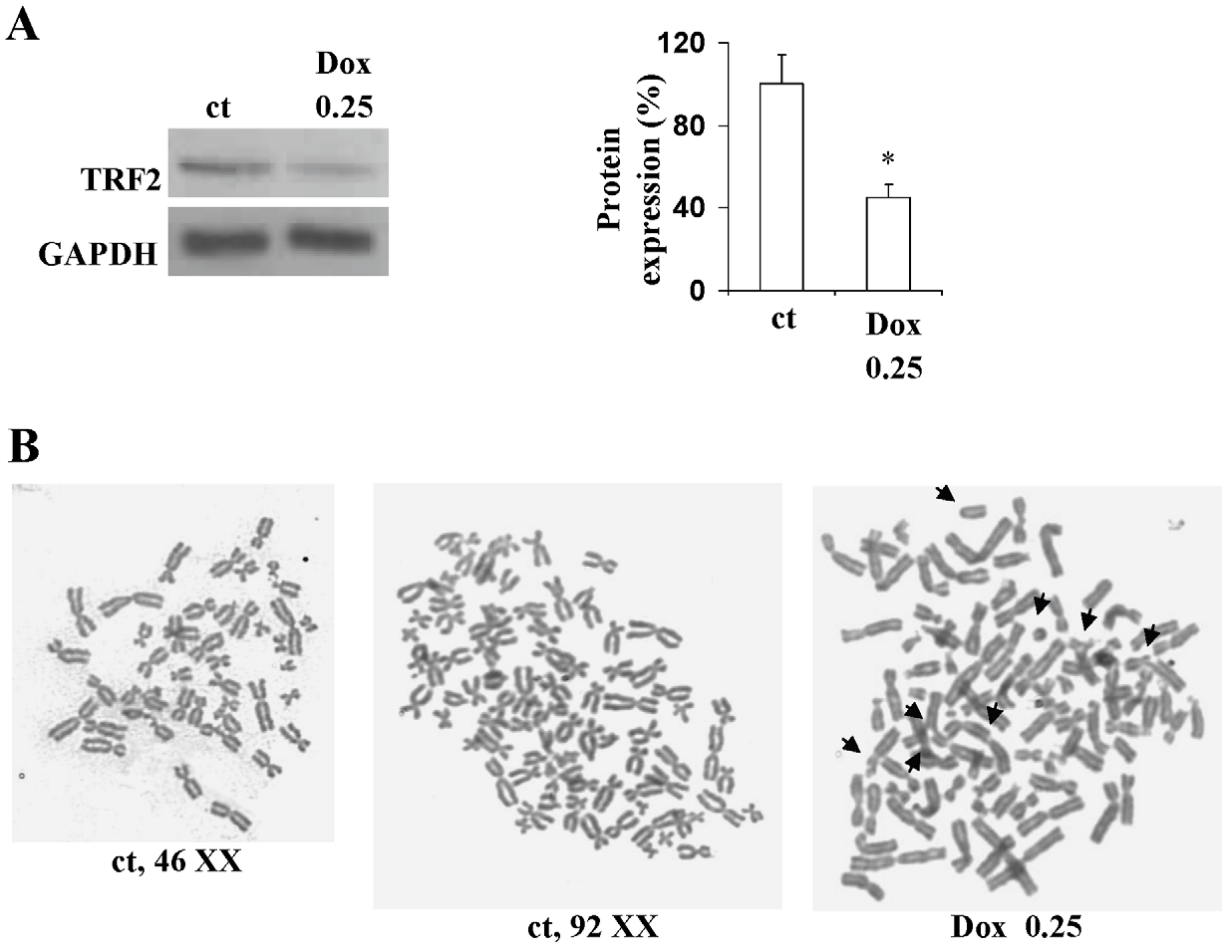
Pulsed incubation of doxorubicin induces telomeric dysfunction and G2 cell cycle arrest. Cells were cultured for 24 h after treatment with doxorubicin 0,25 µM and then analyzed. (A)Western blot analysis of TRF2. (B) Metaphase spreads. Chromosomal abnormalities are indicated by arrows (magnification, ×1000).

**Figure 7:**
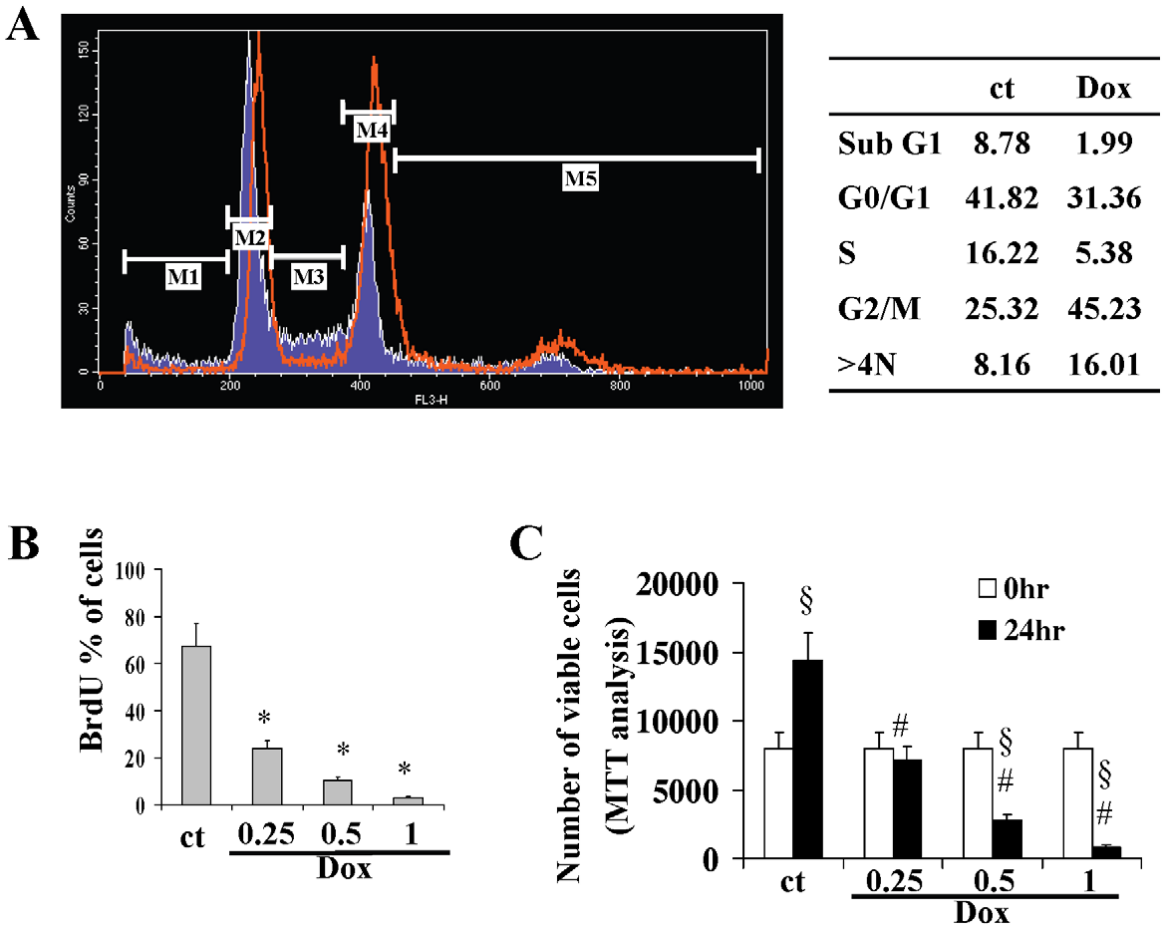
Doxorubicin induces cell cycle alterations and impairs cell viability. (A) Cell cycle analysis: the blue area represents the control cells, the red line indicates treated cells. Cells were cultured for 24 h after treatment with doxorubicin 0,25 µM and then analyzed. Phases of the cell cycle are indicated: Sub G0 [M1], G0/G1 [M2], S [M3], G2/M [M4], >4N [M5]. (B) Cell proliferation evaluated by BrdU incorporation. Cells were cultured for 24 h after treatment with various doses of doxorubicin and then analyzed. BrdU data were normalized to untreated (media alone) cells (C), Number of viable cells evaluated by MTT assay. Cells were analyzed before treatment and 24 hours after treatment with various doses doxorubicin. ct, control; Dox, doxorubicin. *p<0.05 vs ct. § p<0.05 vs 0 h; # p<0.05 vs ct 24 hr.

Recently we demonstrated that low doses of doxorubicin induce senescence in cardiomyocytes by altering the TRF2 levels, thus leading to telomere uncapping and chromosome instability [7]. Pro-senescent doses of doxorubicin also induced these effects in EPCs. Western blot analysis showed a decrease in the protein levels of TRF2 (Fig. 6A), and karyotype analysis revealed chromosomal instability and breakage with structural abnormalities including end-to-end fusions, string, ring and triradial configurations. We observed increases in numerical alterations: 15% of metaphases in the treated cells had tetraploidy (92, XX) versus 8% in untreated cells (Fig. 6B). As shown by FACS analysis of PI–labelled cells, 0.25 µM doxorubicin did not increase the sub-G0 phase that is typical of apoptosis, but it decreased the number of S-phase cells by 66%, caused arrest in G2/M and confirmed the 2-fold increase in hyperploid cells (>4N phase) (Fig. 7A).

In agreement with FACS analysis, mitotic labeling through BrdU, that is incorporated into the DNA of dividing cells during S-phase demonstrated that doxorubicin impairs EPC proliferation which is affected in a dose dependent manner (Fig. 7B). Also MTT that measures mitochondrial metabolic rate documented that doxorubicin decreases viable cell numbers, even though our data show that in comparison with BrdU, MTT assay will provide an underestimation of the antiproliferative effects of doxorubicin (Fig. 7C). This is in agreement with previous studies demonstrating that depending on the agent to be evaluated, MTT based method may result in an underestimation of the antiproliferative efficacy [22].

## Discussion

EPCs have a very low level of replicative senescence. Replicative senescence depends upon the degree of telomere shortening and it occurs when the cell division rate progressively slows following a period of rapid proliferation, and then ultimately ceases altogether. Our experiments document that after brief exposure to sub-apoptotic doses of doxorubicin, EPCs undergo stress-induced premature senescence (SIPS). Unlike replicative senescence, SIPS, which is sometimes also referred to as rapid or accelerated senescence, is caused by telomere dysfunction without overall shortening because the telomere could not be shortened to the threshold length within such a short period of time [23].

We have previously shown that in neonatal rat cardiomyocytes and H9c2 myoblasts [7] sub-apoptotic doxorubicin down-regulates TRF2 whose main role is to maintain the t-loop telomeric structure that governs chromosomal stability. In this study we obtained similar results in EPCs, in which doxorubicin induces TRF2 down-regulation and, as shown by metaphase analysis, chromosomal instability, covalent fusion and mitotic defects with consequential chromosome segregation errors. Upon the next division, misligation of two non-matching ends results in a chromosome with a double strand break at one end that will be fused to another uncapped end, perpetuating a cycle of breakage-fusion-bridge that may lead to late cell death [24], [25].

Previous studies have shown that SIPS is often mediated by p16^INK4A^, the tumor suppression protein which is considered a marker of cellular senescence in a number of stem cell types including neural, hematopoietic, cardiac and pancreas islet progenitor cells [26]–[29].

p16^INK4A^ can bind and inhibit the cyclin dependent kinases 4 and 6 (CDK4 and CDK6), and at the same time it can disrupt the CDK complexes that release other inhibitors, such as p27, which can act by inhibiting CDK2. As a result, p16^INK4A^ increases the expression of the hyperphosphorylated Rb thus blocking the entry of proliferating cells into S phase and inducing a G1 phase arrest that contributes to cellular senescence [30].

We found that doxorubicin 0.25 µM increases the p16^INK4A^ protein levels which are closely associated with the amount of senescent cells, but in agreement with previous studies we did not find that it induced G1 phase arrest, but rather, that it produced cell cycle arrest at G2/M boundary [3], [31]. This result suggests that the doxorubicin-induced increase of p16^INK4A^ does not regulate the cell cycle in this model of SIPS, even though its induction may be associated with other aspects of the senescent phenotype. In this respect, it is noteworthy that p16^INK4A^ accumulates in the cytoplasmic perinuclear zone of doxorubicin-induced senescent cells, but not in cells that become senescent as a result of prolonged confluence in culture. It is likely that the pathways that regulate the senescence process may vary according to the type of stress [29], [32].

Recent findings have demonstrated that p16^INK4A^ is also involved in the control of most events besides cell cycle regulation. Cytoplasmic accumulation may indicate other functional aspects related to specific phenotypes. In lung carcinoma cells it may represent a mechanism of p16^INK4A^ inactivation similar to what is observed in other tumor suppressor genes [33], while in advanced gastric cancer it appears to be a good prognostic indicator and its co-localization with anion exchanger 1 predicts a lack of metastasis [34]. Alhaja demonstrated that cyoplasmic p16^INK4A^ inhibits the spreading and migration in human umbilical vein endothelial cells, and co-localizes with άVβ3 integrin [35]. Our experiments show that besides p16^INK4A^ cytoplasmic localization, the doxorubicin-induced senescent-like phenotype is characterized by a morphologically flattened and enlarged cell shape, an increase in SA-b-gal activity and the structural alteration and redistribution of F-actin. This cytoskeleton remodeling is associated with remarkable impairment of EPC migration.

The MAPK signalling that converges on JNK and p38 plays an important role in doxorubicin-induced senescence and apoptosis in many cell types [7], [36]. A number of studies have also established the involvement of MAPKs in stem cell senescence. Ito and colleagues [37] demonstrated that p38 regulates senescence and p16^INK4A^ in hematopoietic stem cells, Zhang [38] showed that inhibiting the p38 pathway mitigates both senescence and the increase in p16^INK4A^ expression induced by TNF-ά in human umbilical cord blood EPCs.

We observed that pre-treatment with a specific inhibitor of p38 reduces the number of SA-b-gal positive cells, decreases the accumulation of p16INK4A and almost completely prevents the structural changes of the F-actin fibers induced by doxorubicin. On the contrary, pre-treatment with the specific inhibitor of JNK decreases senescence but induces apoptosis, as evidenced by the increase in ssDNA positivity and destruction of F-actin stress fibers.

The balance between JNK and p38 activity in defining cell fate in response to stress has already been demonstrated by other groups [39] and by ourselves [16].

Present data confirm that p38 and JNK pathways antagonistically control cellular senescence, p16^INK4A^ expression and structure of F-actin stress fibers in EPCs treated with doxorubicin. JNK activation promotes cell survival by countering the negative effects that doxorubicin induces via p38 activation.

Induction of oxidative stress is one of the mechanisms through which doxorubicin acts. Oxidative stress appears to activate the senescence program mainly by involving the p16^INK4A^-pRb pathway through the mediation of the p38 signalling cascade [40], [41]. It is possible that in EPCs, ROS accumulation, induction of p38 and JNK signalling that regulate p16^INK4A^, together with telomere dysfunction, may jointly contribute to doxorubicin induced senescence. In fact, NAC, an antioxidant, down-regulates SA-b-gal positivity and p16^INK4A^ levels, and sustains the hypothesis that NAC acts by inhibiting the signal that is likely induced by oxidative stress. Consistent with this finding, previous reports [9], [42] have indicated that pre-treatment with antioxidants contrasts signalling transduced by p38 and JNK doxorubicin-treated cells.

It has been shown that when the heart is exposed to doxorubicin several mechanisms are activated in an attempt to promote cell protection and tissue repair. The neuregulin/erbB2 system is probably the most important survival pathway to be activated in response to doxorubicin [43]. The high cardiotoxic effects of trastuzumab, an anti-erbB2 humanized monoclonal antibody, which has been observed in erbB2+ breast cancer patients that have been pre-treated or co-treated with anthracyclines, depend on the loss of this survival pathway [16], [44]. EPCs also have the potential for heart protection or repair in the course of anthracycline treatment. A number of studies have shown that EPCs are able to proliferate and differentiate into mature endothelial cells [45] and to secrete paracrine factors that prevent apoptosis of mature endothelial cells and induce hypertrophy of cardiac muscle cells [46], [47]. There is also evidence that EPCs may trans-differentiate into cells of the myocardial lineage [48]. This EPC function is of great interest in the light of the recent study by Huang [5] who found that both reduced capillary density and reduced vascular endothelial growth factor expression in anthracycline-treated hearts limit the ability of the cardiac tissue to respond to further stress. Hamed, furthermore, found that doxorubicin targets EPCs thereby reducing the number of cells and impairing their function [4].

The present study takes a step forward in understanding doxorubicin cardiomyopathy. First, it shows that pulsed exposure to low sub-apoptotic doses of doxorubin induces premature senescence in EPCs, and it provides us with insight into the mechanisms of doxorubicin-induced senescence, which is needed to further explore strategies to protect EPCs.

Secondly, it emphasizes the importance of cardiomyocyte senescence as a major mechanism of doxorubicin cardiomyopathy [6]. Even though senescence may be viewed as less severe than apoptosis, present findings suggest that as compared to apoptotic cardiomyocytes, senescent ones produce or secrete less homing factors that would attract EPCs to the site of injury thus impairing the heart's ability to limit the damage.
